# Nurses in Iran: A Force for Change

**DOI:** 10.5812/nms.14726

**Published:** 2013-12-10

**Authors:** David C Benton

**Affiliations:** 1 International Council of Nurses, Geneva, Switzerland

**Keywords:** Nursing, Social Change, Cooperative Behavior

I want to say a few words about perspectives of nursing in Iran and its place in the international family. I see nurses in Iran as a dynamic force of change. Three things can be discussed here:

1. Nurses here in Iran;
2. Nurses as a bridge to local and global communities; and
3. The role of the Iranian nursing organization.

Whilst I will highlight some of the excellent things I have seen in Iran, I will also point out the opportunities for further progress. So what can I say about nursing here in Iran. First, Iranian nursing community has many things to be proud of. Iranian nurses are competent, enthusiastic and innovative. I have had the privilege to work with a group of thirty-two nurses for the past two years. They have embraced new approaches to learn and rise the challenge of working in teams to tackle critical issues in the healthcare services. They have been focused and honest about the need to further development of community based services in Iran (1). Because of this issue, I will briefly say a few more words about one of the team projects. A group of nurse leaders have worked to identify how the primary and community care services can be improved for the citizens of Iran. The Family Health Nurse has a complimentary role to the existing system and extends its reach. It does not replicate the family physician role but instead provides a health rather than illness focused service to families. A service that can capitalize on the trust that the profession enjoys and the autonomy that well trained nurses must be granted. The evidence clears the cost-effective roles that the citizens of Iran can fundamentally benefit from the development and implementation of this role (2). The opportunity is there. You have expert nurses that can work with the Ministry of Health from analysis to implementation. I hope to see rapid progress. This is the challenge I give you all. Iranian citizens deserve all our best efforts in this regard. The trends to the future of nursing education also should be addressed. Some of these trends are as follows:

- The explosion of technology,
- Globalization,
- The era of the educated consumer, alternative therapies, genomics, and palliative care,
- A shift to population-based care and the increasing complexity of patient care,
- Health care costs and challenges posed by managed care,
- The impact of health policy and regulation,
- A growing need for interdisciplinary education and collaborative practice,
- The growing nursing shortage along with the opportunities for lifelong learning and workforce development,
- Significant advances in nursing science and research (3).

Let me turn now to the role that nurses can play as a bridge to local and global communities. I am very excited about the development of a screening program for citizens delivered by nurses under the auspices of the Iranian Nursing Organization (2). This is a bold and innovative move. It is unique in the world and it demonstrates the commitment of the Iranian Government to tackle the increasing burden of non-communicable diseases that will cripple health systems around the world. Iran is leading the way and should be recognized for this creative solution where nurses provide a bridge into local communities. Nurse can be found in all areas of Iran. They can work with the most marginalized communities. I challenge you all to implement quickly this initiative. Work diligently with public health colleagues to measure the differences in terms of the quality of lives and years saved. Do not miss this opportunity and teach other nations how this global scourge can be addressed. There are 19 million nurses in the world (4). When I came to work with nurses here in Iran I am ashamed to say the image I had of your country was that promoted in the popular western media. The situation could not have been more different. You have a beautiful country, a friendly and sincere culture and I am delighted to have had this opportunity to work so closely with your nurses. They are true ambassadors for your country and they must be encouraged to share their stories of the realities of Iran just as I did in a widely read journal that talked about a range of countries where nurses were doing exceptional things and working with their governments to achieve positive change, so more nurses need to be encouraged to write in the international journals. And more nurses need to be supported to attend international conferences to play their part in providing a balanced view of nursing, care and life here in Iran. As I said in my article in the Online Journal of Issues in Nursing the Iranian Nursing Organization has achieved an enormous improvement in a relatively short time (5). They are active across the country to reach out to their colleagues in other parts of the world and importantly they work regularly with all forms of media here in the country. But with the agreement of government they could do more. Esmaeili et al. has evaluated the common challenges facing the nursing associations in Iran. Insufficient supports from the associations, financial problems, non-professional activities of associations and lack of interactions among associations are the important challenges that the nursing associations confront within Iran (6). These challenges need to be addressed in future. A countrie's human resources are one of its most valuable assets. I would like to see the Iranian Nursing Organization to have a much important role in the registration of nurses and as a consequence provides the basis for coherent workforce planning and capacity development. Nurses in the world and nurses in Iran are confronting with some common challenges including: The nursing shortages, job dissatisfaction, and poor social position of nurses, the gap between theory and practice and lack of community-based nursing care (7). ICN stands ready to work with nurses in Iran to address these and other challenges. Together nurses are a force for change.
